# Supplementary material for: Hydrodynamic slip can align thin nanoplatelets in shear flow
Source: Nat Commun. 2020 May 15;11:2425. doi: 10.1038/s41467-020-15939-w (PMC7229003; doi:10.1038/s41467-020-15939-w)
Supplement: Supplementary file 1 — Supplementary Information [file 41467_2020_15939_MOESM1_ESM.pdf]

Supplementary Information
Hydrodynamic slip can align thin nanoplatelets in shear flow

Catherine Kamal[1], Simon Gravelle[1,2], Lorenzo Botto[1,3]
[1] *School of Engenieering and Material Science, Queen Mary University of London, UK*
[2] *Facultad de Ingeniería y Ciencias, Universidad Adolfo Ibáñez, Viña del Mar, Chile*
[3] *Process & Energy Department, Faculty of Mechanical, Maritime and Materials Engineering, Delft University of Technology, Leeghwaterstraat 39, 2628CB Delft, The Netherlands*
(Dated: March 17, 2020)

Supplementary Figures 1-5
Supplementary Method
Supplementary Notes 1-2

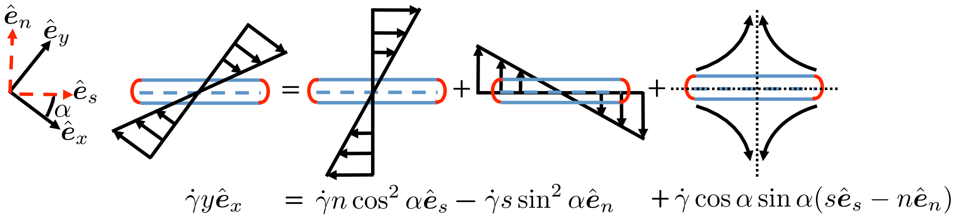

Supplementary Figure 1: **Sketch of the flow field.** Sketch of the flow field around a 2D platelet in an external shear flow $\boldsymbol{u}_\infty = \dot{\gamma} y \hat{\boldsymbol{e}}_x$.

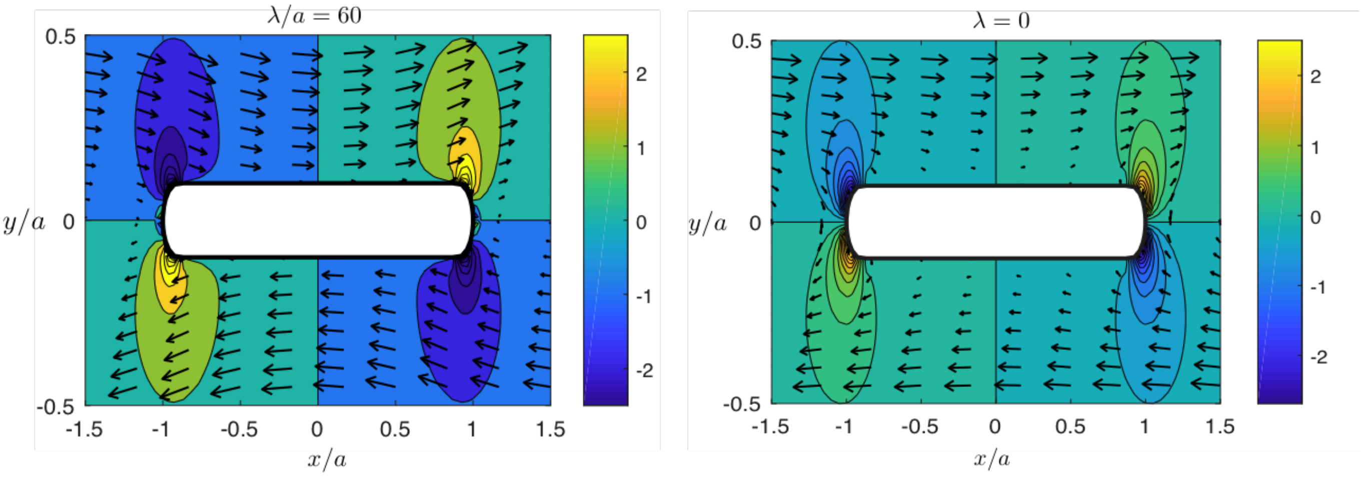

Supplementary Figure 2: **Velocity and pressure field.** Velocity field (arrows) and normalised pressure (colour) around a platelet, with and without slip. Both flow and pressure fields are calculated directly by the BIM method [1]. The pressure is normalised by the shear stress $\eta\dot{\gamma}$. As expected the flow fields obtained with and without slip are different: with slip, the flow has a finite value at the slender surface of the body, and with no slip the magnitude of the flow is zero over the slender surface. The pressure field is is qualitatively similar in the two cases, presenting a large pressure gradient near the edges. However, a larger pressure gradient exists at these locations in the presence of slip.

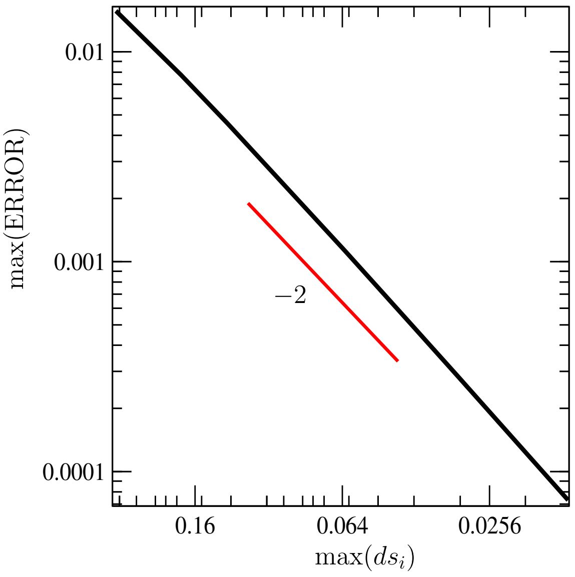

Supplementary Figure 3: **Computational error.** Maximum error between the hydrodynamic traction computed from BI and the exact solution (19) vs maximum grid spacing for an infinite cylinder of radius $R = 1$ in a shear flow of strength $\dot{\gamma}\eta = 1$. The slip length is $\lambda = 1$.

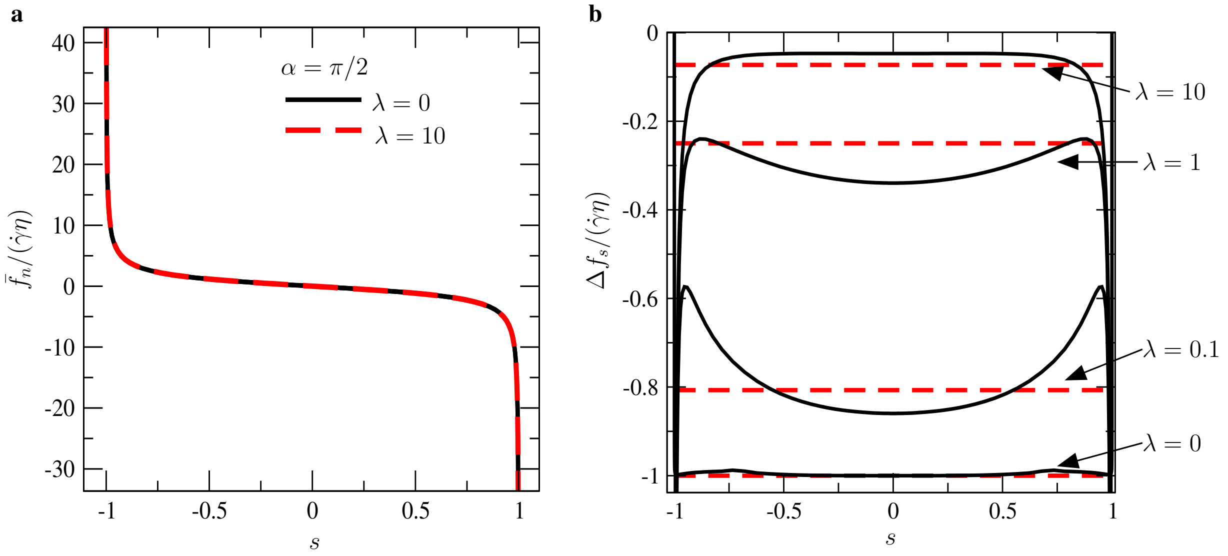

Supplementary Figure 4: **Hydrodynamic traction for** $\alpha = \pi/2$**.** BI simulations of the non-dimensional hydrodynamic traction for different values of λ and for $\alpha = \pi/2$. The surface S is an ellipse with $a/b = 100$. **a** $\bar{f}_n$ is independent of λ to leading order. **b** $\Delta f_s \to 0$ as $\lambda/a \to \infty$. Black line, BI; red dashed line, Eq. (27).

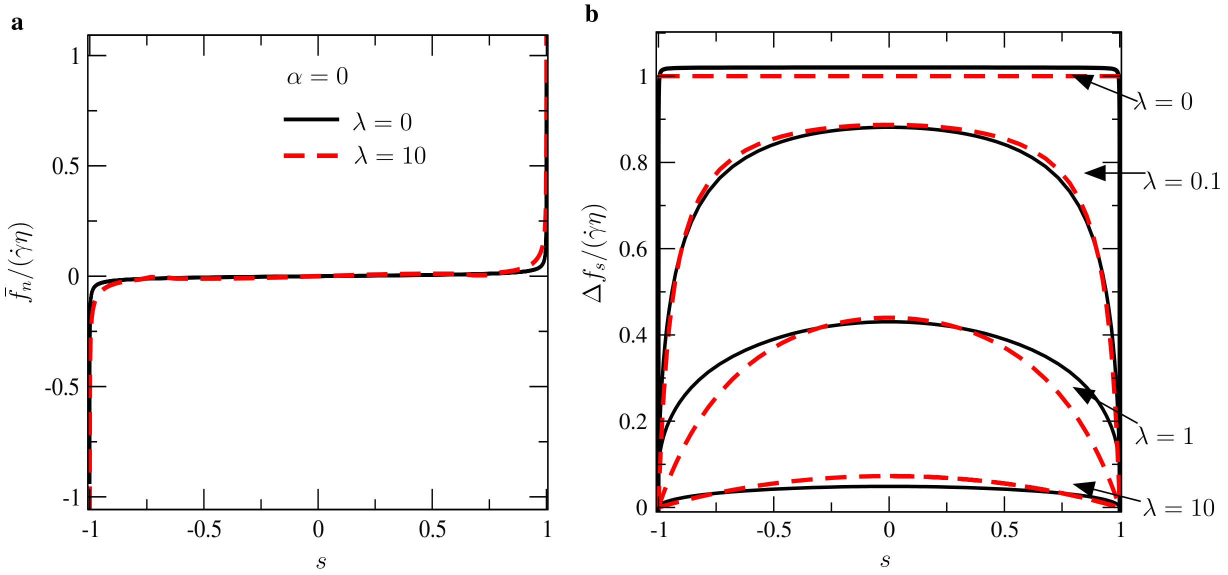

Supplementary Figure 5: **Hydrodynamic traction for** $\alpha = 0$**.** BI simulations of the non-dimensional hydrodynamic traction for different values of λ and for $\alpha = 0$. The surface S is an ellipse with $a/b = 100$. **a** $\bar{f}_n$ is independent of λ to leading order. **b** Δf_s decreases as λ increases. Black line, BI; red dashed line, Eq. (30) without the contribution form the edge.

Supplementary Method

This Supplementary Method presents details concerning the Boundary Integral formulation.

General formulation. The hydrodynamic torque on a body is given by the surface integral of the moment of the hydrodynamic traction $\boldsymbol{f}$ with respect to the geometric centre of the body

$$\boldsymbol{T} = \int_S \boldsymbol{f} \times \boldsymbol{x} \, \mathrm{d}S. \tag{1}$$

In Stokes flows, an accurate method to calculate the hydrodynamic traction is the Boundary Integral (BI) method [1]. In this method the fluid velocity at a point $\boldsymbol{x}_1$ on the surface S of a body $\mathcal{B}$ is related to the traction force $\boldsymbol{f}$ on this boundary through the following integral equation:

$$\int_S \boldsymbol{n} \cdot \boldsymbol{K} \cdot \boldsymbol{u}^{\mathrm{sl}} \mathrm{d}S - \frac{1}{\eta} \int_S \boldsymbol{G} \cdot \boldsymbol{f} \, \mathrm{d}S = \frac{\boldsymbol{u}^{\mathrm{sl}}(\boldsymbol{x}_1)}{2} - \boldsymbol{u}_\infty(\boldsymbol{x}_1), \tag{2}$$

where $\boldsymbol{n}$ is the surface normal and $\boldsymbol{u}_\infty$ is the external velocity field. The second-order tensors $\boldsymbol{G}$ and the third-order tensor $\boldsymbol{K}$ are the tensors associated with the 'stokeslet' and 'stresslet' (the velocity and stress field generated by a point force) [1]. In the formulation above we have allowed for a slip velocity $\boldsymbol{u}^{\mathrm{sl}}$ at the boundary. Assuming a Navier slip equation, the slip velocity is closed in terms of the traction by using

$$\boldsymbol{u}^{\mathrm{sl}} = \frac{\lambda}{\eta} \boldsymbol{n} \times \boldsymbol{f} \times \boldsymbol{n}, \tag{3}$$

where λ is the slip length [2–4].

Formulation for a symmetric 2D platelet in an external shear flow field. We consider a two-dimensional body that is symmetric about two orthogonal planes intersecting at the body's geometric centre (**Supplementary Figure 1**). In this case, the surface S is actually a closed line, and so can be parameterised as $S = \{s\hat{\boldsymbol{e}}_s \pm h(s)\hat{\boldsymbol{e}}_n : -a \le s \le a\}$. Using the 2D 'stokeslet's' and 'stresslet's' tensors [1],

$$G_{ij}(s,h) = (4\pi)^{-1} \left(-\partial_{ij} \ln \sqrt{s^2+h^2} + 2x_i x_j/(s^2+h^2) \right), \quad K_{ijk}(s,h) = -(\pi)^{-1} \left(x_i x_j x_k/(s^2+h^2)^4 \right), \tag{4}$$

respectively, Eq. (2) can be written as

$$\int_S \boldsymbol{n} \cdot \boldsymbol{K}(s',h') \cdot \boldsymbol{u}^{\mathrm{sl}} \mathrm{d}S - \frac{1}{\eta} \int_S \boldsymbol{G}(s',h') \cdot \boldsymbol{f} \mathrm{d}S = \frac{\boldsymbol{u}^{\mathrm{sl}}(\boldsymbol{x}_1)}{2} - \boldsymbol{u}_\infty(\boldsymbol{x}_1), \tag{5}$$

where $s' = s - s_1$, $h' = h(s) - h(s_1)$, and $\mathrm{d}S = \sqrt{||\partial_s \boldsymbol{x}||}\mathrm{d}s_1$. For an external shear flow $\boldsymbol{u}_\infty = \dot{\gamma} y \hat{\boldsymbol{e}}_x$. This equation can be decomposed into a normal component (in the direction $\hat{\mathbf{e}}_n$)

$$\int_S \hat{\mathbf{e}}_n \cdot \left(\boldsymbol{n} \cdot \boldsymbol{K}(s',h') \cdot \boldsymbol{u}^{\mathrm{sl}} \right) \mathrm{d}S - \frac{1}{\eta} \int_S \hat{\mathbf{e}}_n \cdot \boldsymbol{G}(s',h') \cdot \boldsymbol{f} \mathrm{d}S = \frac{u_n^{sl}}{2} - u_{\infty,n}, \tag{6}$$

and a tangential component to s (in the direction $\hat{\mathbf{e}}_s$)

$$\int_S \hat{\mathbf{e}}_s \cdot \left(\boldsymbol{n} \cdot \boldsymbol{K}(s',h') \cdot \boldsymbol{u}^{\mathrm{sl}} \right) \mathrm{d}S - \frac{1}{\eta} \int_S \hat{\mathbf{e}}_s \cdot \boldsymbol{G}(s',h') \cdot \boldsymbol{f} \mathrm{d}S = \frac{u_s^{sl}}{2} - u_{\infty,s}. \tag{7}$$

The components of the external shear flow are $u_{\infty,n} = -\dot{\gamma}(s \sin^2\alpha + n \sin\alpha\cos\alpha)$ and $u_{\infty,s} = \dot{\gamma}(n\cos^2\alpha + s\sin\alpha\cos\alpha)$. In this frame of reference the surface S can be decomposed into its upper surface $S^+ = \{(s, h(s)) : -a \le s \le a\}$ and its lower surface $S^- = \{(s, -h(s)) : -a \le s \le a\}$. These surfaces S^+ and S^- are mirror symmetric with respect to the centreline $n = 0$. To exploit the fact that S^+ and S^- are mirror symmetric with respect to the centreline, we introduce a decomposition of the surface fields into a symmetric and anti-symmetric components. Given a generic vector of component p_i, the antisymmetric part of p_i is

$$\mathrm{Asym}\{p_i(s,h)\} = \Delta p_i(s,h) = \frac{p_i(s,h) - p_i(s,-h)}{2}. \tag{8}$$

and the symmetric part of p_i is

$$\mathrm{Sym}\{p_i(s,h)\} = \bar{p}_i(s,h) = \frac{p_i(s,h)+p_i(s,-h)}{2}. \tag{9}$$

We apply these operators to the normal and tangential components of the vector boundary integral Eq. and obtain the following 4 scalar equations: Symmetric part, normal component:

$$S_n[\Delta f_s, \bar{f}_n] + K_n[\Delta u_s^{\mathrm{sl}}, \bar{u}_n^{\mathrm{sl}}, \lambda] \;=\; \frac{\bar{u}_n^{\mathrm{sl}}}{2} + \dot{\gamma} s \sin^2\alpha, \tag{10}$$

Symmetric part, tangential component:

$$S_s[\bar{f}_s, \Delta f_n] + K_n[\bar{u}_s^{\mathrm{sl}}, \Delta u_n^{\mathrm{sl}}, \lambda] \;=\; \frac{\bar{u}_s^{\mathrm{sl}}}{2} - \dot{\gamma} s \sin\alpha\cos\alpha. \tag{11}$$

Antisymmetric part, normal component:

$$S_n[\bar{f}_s, \Delta f_n] + K_s[\bar{u}_s^{\mathrm{sl}}, \Delta u_n^{\mathrm{sl}}, \lambda] \;=\; \frac{\Delta u_n^{\mathrm{sl}}}{2} + \dot{\gamma} h \sin\alpha\cos\alpha. \tag{12}$$

Antisymmetric part, tangential component:

$$S_s[\Delta f_s, \bar{f}_n] + K_s[\Delta u_s^{\mathrm{sl}}, \bar{u}_n^{\mathrm{sl}}, \lambda] \;=\; \frac{\Delta u_s^{\mathrm{sl}}}{2} - \dot{\gamma} h \cos^2\alpha. \tag{13}$$

The integrals S_s and S_n are defined as

$$S_s[\Delta f_s, \bar{f}_n] = -\frac{1}{\eta}\int_{S^+} \Big(G_{ss}(s',h') - G_{ss}(s',\hat{h})\Big)\,\Delta f_s + \Big(G_{sn}(s',h') + G_{sn}(s',\hat{h})\Big)\,\bar{f}_n \mathrm{d}S, \tag{14}$$

and

$$S_n[\Delta f_s, \bar{f}_n] = -\frac{1}{\eta}\int_{S^+} \Big(G_{nn}(s',h') + G_{nn}(s',\hat{h})\Big)\,\bar{f}_n + \Big(G_{sn}(s',h') + G_{sn}(s',\hat{h})\Big)\,\Delta f_s \mathrm{d}S, \tag{15}$$

respectively, where $\hat{h} = -h(s) - h(s_1)$. The integrals K_s and K_n are defined as

$$K_i[\Delta u_s, \bar{u}_n, \lambda] = \frac{1}{\eta}\int_{S^+} \left[n_s \Delta u_s(\lambda)\left(K^-_{inn} - K^-_{iss}\right) + \left(n_n \Delta u_s(\lambda) + n_s \bar{u}_n(\lambda)\right) K^+_{isn}\right] \mathrm{d}S, \tag{16}$$

where $i = \{s, n\}$, $\boldsymbol{K}^+ = \boldsymbol{K}(s',\hat{h}) + \boldsymbol{K}(s',\hat{h})$ and $\boldsymbol{K}^- = \boldsymbol{K}(s',\hat{h}) - \boldsymbol{K}(s',\hat{h})$. Eq. (10) and Eq. (13) are equations for the hydrodynamic traction Δf_s and $\bar{f}_n$, due to the 'shear' components of the external flow field $\boldsymbol{u}_{\infty,S} = \dot{\gamma}\left(h\cos^2\alpha\hat{\boldsymbol{e}}_s - s\sin^2\alpha\hat{\boldsymbol{e}}_n\right)$. Eq. (12) and Eq. (11) are equations for Δf_n and $\bar{f}_s$ due to the 'extensional' components of the external flow field $\boldsymbol{u}_{\infty,E} = \dot{\gamma}\left(s\cos\alpha\sin\alpha\hat{\boldsymbol{e}}_s - h\cos\alpha\sin\alpha\hat{\boldsymbol{e}}_n\right)$ (**Supplementary Figure 1**). Due to symmetry properties of the external flow field with respect to the rigid body, the hydrodynamic traction components appearing in Eq. (12) and Eq. (11) due to an extensional external flow field give zero hydrodynamic torque [5]. For a given inclination angle α, equations (10) and (13) can be used to calculate the traction components contributing to the hydrodynamic torque $T_\lambda(\alpha)\hat{\boldsymbol{e}}_z$. It is only necessary to calculate the total torque $T_\lambda(\alpha)$ for two orthogonal angles, $\alpha = 0$ and $\alpha = \pi/2$. The torque for other orientations can be calculated from $T_\lambda(\alpha) = \cos^2\alpha T_\lambda(0) + \sin^2\alpha T_\lambda(\pi/2)$. Once $\boldsymbol{f}$ is known over the surface, then the flow field and pressure gradient can also be calculated [1], as shown for example in **Supplementary Figure 2** for a flow field near a platelet held aligned with the flow with slip (left) and without slip (right).

Numerical implementation and validation. To solve the set of equations (10 to 13) we use the numerical method described in Ref. [6], suitably modified to account for the slip velocity [3, 4]. This method involves discretising the traction and slip velocity as N piece wise-continues constants $\{\boldsymbol{f}[1]\ldots\boldsymbol{f}[N]\}$ and $\{\boldsymbol{u}^{\mathrm{sl}}[1]\ldots\boldsymbol{u}^{\mathrm{sl}}[N]\}$, respectively over each element of the surface $S_i = \int_{s_i}^{s^{i+1}} \mathrm{d}S$. Under this discretisation, the discretised BI Eq. (5) becomes, for each point $s[j]$ for $j = \{1\ldots N\}$,

$$\boldsymbol{u}_\infty[s[j]] = \sum_{i=1}^{N}\left[\frac{1}{\eta}\boldsymbol{f}[i]\int_{s_i}^{s_{i+1}} \boldsymbol{G}(s, s[j])\mathrm{d}S(s) + \boldsymbol{u}^{\mathrm{sl}}[i]\int_{s_i}^{s_{i+1}} \boldsymbol{K}(s, s[j])\cdot\boldsymbol{n}(s)\mathrm{d}S(s)\right]. \tag{17}$$

Each sub-integral is evaluated using Gaussian's quadratic weights [6]. If $i = j$, then the sub-integrals are singular. The singular sub-integrals are evaluated analytically, by Taylor expanding about the singular points. Using the closed relation for the slip velocity with the traction Eq. (3), the discretised Eq. (17) can be arranged into a system of N linear equations for the traction $\boldsymbol{f}[j]$. This system of equations is solved for $\boldsymbol{f}[j]$ using Gaussian elimination [7].

For validation, we compared against the exact solution for the hydrodynamic stress for shear flow past an infinite cylinder with axis parallel to the vorticity direction [5]. The solution was developed for the no slip case. We have extended it to the more general case of a Navier slip boundary condition. The velocity disturbance induced by the cylinder can be expressed as a linear combination of a rotlet, a stresslet and a potential quadrupole, all the singularities being located at the cylinder's centre. Hence, the Cartesian coordinates of the velocity field can be written as:

$$u_x = \dot{\gamma} y + c_1 \frac{-y}{r^2} - c_2 \frac{2x^2 y}{r^4} + c_3 \frac{\partial^3}{\partial x^2 \partial y} \ln r, \quad u_y = c_1 \frac{x}{r^2} - c_2 \frac{2y^2 x}{r^4} - c_3 \frac{\partial^3}{\partial y^2 \partial x} \ln r, \quad r = \sqrt{x^2 + y^2}, \tag{18}$$

where a, b and c are constants. Imposing that the flow velocity satisfies a Navier slip boundary condition with slip length λ on the cylinder's surface $r = R$, gives

$$c_1 = \frac{\dot{\gamma} R^3}{2(R + 2\lambda)}, \quad c_2 = \dot{\gamma} R^2 \frac{R + 2\lambda}{R + 4\lambda}, \quad c_3 = \frac{\dot{\gamma} R^5}{4(R + 4\lambda)}.$$

Introducing the polar angle θ, the normal and tangential components of the hydrodynamic traction and the total torque are readily calculated as :

$$\begin{aligned} \bar{f}_n &= \dot{\gamma}\eta \frac{\cos\theta \left(R(R + 24\lambda) + 48\lambda^2 - 24\lambda(R + 2\lambda)\cos\theta^2\right)}{(R + 2\lambda)(R + 4\lambda)}, \\ \Delta f_s &= \dot{\gamma}\eta \frac{3\sin\theta \left(8\lambda(R + 2\lambda)\cos\theta^2 + R(R + 8\lambda/3)\right)}{(R + 2\lambda)(R + 4\lambda)}, \\ T &= -w\eta 4\pi c_1 = -w\eta\dot{\gamma} \frac{2R^3\pi}{R + 2\lambda}. \end{aligned} \tag{19}$$

Supplementary Figure 3 shows the maximum difference between the numerical and exact values of $\bar{f}_n$ and Δf_s for different values of the maximum grid spacing $\max(\mathrm{d}s_i)$. The results show that, owing to the piece-wise continuous constant traction function adopted, the error converges as $\propto \max(\mathrm{d}s_i)^{-2}$.

Supplementary Note 1

This supplementary note presents details concerning the asymptotic analysis of the hydrodynamic traction.

2D plate. For a generic tensor $\boldsymbol{A}$, the surface integral of $\boldsymbol{f}\cdot\boldsymbol{A}$, to leading terms $O(b)$ about the centreline, satisfies

$$\int_{S^+} \boldsymbol{f}\cdot(\boldsymbol{A}(s',h')\pm\boldsymbol{A}(s',\hat{h}))\mathrm{d}S \approx$$
$$\int_{-a}^{a} \boldsymbol{f}\cdot\left[(\boldsymbol{A}(s',0)\pm\boldsymbol{A}(s',0)) + \left(h'\partial'_h\boldsymbol{A}(s',h')|_{h'=0} \pm \hat{h}\partial_{\hat{h}}\partial h\boldsymbol{A}(s',\hat{h})|_{\hat{h}=0}\right)\right]\mathrm{d}s_1$$
$$+\boldsymbol{f}\cdot\int_{-a}^{a}\left[\boldsymbol{A}(s',0)\pm\boldsymbol{A}(s',-2h)\right]\mathrm{d}s_1. \tag{20}$$

We have used this formula, which is simply a Taylor series expansion about the centreline up to terms of $O(b)$) to approximate the integrals in (10) and (13). For $S_s[\Delta f_s,\bar{f}_n]$ and $S_n[\Delta f_s,\bar{f}_n]$ we obtain

$$S_s \simeq -h\Delta f_s + 2\int_{-a}^{a}\frac{\bar{f}_n(s_1)h(s_1)s'}{s'^2+4h'^2}\mathrm{d}s_1 - h\epsilon_s, \tag{21}$$

and

$$S_n \simeq -\frac{h\Delta f_s}{2\pi}\int_{-a}^{a}\frac{1}{s'}\mathrm{d}s_1 + 2\int_{-a}^{a}\left[\bar{f}_n(s_1)\ln(s'^2+4h'^2) - \partial_s(h(s_1)\Delta f_s(s_1))\ln s'^2\right]\mathrm{d}s_1 - h\epsilon_n, \tag{22}$$

where the vector $\boldsymbol{\epsilon}$ of components $\epsilon_s(s,\lambda)$ and $\epsilon_n(s,\lambda)$, represents the contribution of the edges to the integral (this vector tends to zero as $|s\pm a|$ increases [8]). The corresponding approximations for $K_s[\lambda\Delta f_s/\eta, 0, \lambda] = (\lambda/\eta)\int_{-a}^{a}\Delta f_s K^+_{ssn}\mathrm{d}s_1$ and $K_n[\lambda\Delta f_s/\eta, 0, \lambda] = (\lambda/\eta)\int_{-a}^{a}\Delta f_s K^+_{nns}\mathrm{d}s_1$ are

$$\begin{aligned} K_s &= -\frac{\lambda\Delta f_s}{\pi\eta}\left(\frac{2h(s_1)s_1}{s_1^2+4h^2(s_1)} + \frac{1}{2}\arctan\left(\frac{-s_1}{2h(s_1)}\right)\right)\Bigg|_{s_1=-a-s}^{s_1=a-s} - \frac{2\lambda}{\pi\eta}\left(\int_{-a}^{a}\frac{h(s_1)\Delta f_s(s_1)}{s'^2}ds_1 + \Delta f_s h\int_{-a}^{a}\frac{1}{s'^2}ds_1\right) + O(\lambda b^3), \\ &= \frac{\lambda}{\eta}\Delta f_s\left(\frac{1}{2} - \frac{4ah}{\pi(a^2-s^2)} + h\epsilon_n\right) + O(\lambda b^2), \end{aligned} \tag{23}$$

and

$$K_n = \frac{\lambda}{\eta}\left[\frac{4\Delta f_s}{\pi}\left(\frac{h^2(s_1)}{s_1^2+4h^2(s_1)}\right)\Bigg|_{s_1=-a-s_1}^{s_1=a-s_1} + \frac{4h}{\pi}\int_{-a}^{a}\frac{\Delta f_s(s_1)h(s_1)}{s'^3}ds_1 + \frac{4\Delta f_s(s_1)h^2(s)}{\pi}\int_{-a}^{a}\frac{1}{s'^3}ds_1 + h\epsilon_n + O(\lambda b^3)\right]. \tag{24}$$

To derive these equations we have used the fact that $\boldsymbol{n} = n_s\hat{\boldsymbol{e}}_s + n_n\hat{\boldsymbol{e}}_n \approx \hat{\boldsymbol{e}}_n$, $u_n^{\mathrm{sl}} \approx 0$ and $u_s^{\mathrm{sl}} \approx \frac{\lambda}{\eta}\Delta f_s$ away from the edges.

An expansion similar to that in Eq. (21) and Eq. (22) was developed in Ref. [8] for a thin, three-dimensional axisymmetric disk. We found that S_s in that paper is identical to ours to leading order, and S_n differs only by a numerical prefactor. This similarity is a consequence of the velocity in the z (depth) direction being negligible in both problems, to leading order [8]. Thus, as long as the depth of the platelet is large compared to the thickness, the total torque per unit length produced by a given cross-section of a three-dimensional body is the same - up to a numerical prefactor - to the corresponding one for the two-dimensional body. An important observation is that the integral K_s produces a leading-order term that cancels out exactly with the slip velocity $u_s^{\mathrm{sl}} = (\lambda/\eta)\Delta f_s/2$, resulting in a term comparable to S_s. Also, $K_n \ll S_n$ to leading order, so K_n does not affect the value of the tangential traction to this order.

Finding α_c requires calculating the hydrodynamic traction components $\bar{f}_n$ and Δf_s for two angles: $\alpha = \pi/2$ and $\alpha = 0$. We examine the two cases separately.

Case $\alpha = \pi/2$. Inserting the leading-order approximations for the Green's functions into Eq. (10) and Eq. (13) yields

$$0 = h\Delta f_s + \lambda\frac{4ha}{\pi(a^2-s^2)}\Delta f_s + 2\int_{-a}^{a}\frac{\bar{f}_n(s_1)h(s_1)s'}{s'^2+4h'^2}\mathrm{d}s_1 + h\epsilon_s, \tag{25}$$

$$-\dot{\gamma}\eta s = -2\int_{-a}^{a}\bar{f}_n(s_1)\ln(s'^2+4h'^2)\mathrm{d}s_1 + h\epsilon_n. \tag{26}$$

Calculating the integrals above exactly is challenging. We therefore provide the following estimate. Because $K_n \ll S_n$, Eq. (26) is independent of λ at leading order $O(b)$. Hence, f_n is also independent of λ to this order. Moreover, balancing the $O(b)$ terms in Eq. (26) away from the edges gives $f_n \sim O(\dot{\gamma}\eta)$, as confirmed in **Supplementary Figure 4 a**. Eq. (25) instead depends on λ to leading order:

$$\Delta f_s \approx -\frac{1}{1+\frac{4\lambda a}{(\pi(a^2-s^2))}}\left(\frac{2}{h}\int_{-a}^{a}\frac{\bar{f}_n(s_1)h(s_1)s'}{s'^2+4h'^2}\mathrm{d}s_1-\epsilon(s,\lambda)\right)\propto -\frac{\eta\dot{\gamma}}{1+4\lambda/\pi a}. \tag{27}$$

Here the scale '$\dot{\gamma}\eta$' is due to both the terms $\bar{f}_n$ and ϵ_s scaling with $\dot{\gamma}\eta$. The fact that Δf_s vanishes as $\lambda/a \to \infty$ is confirmed in **Supplementary Figure 4 b**. Because $s\bar{f}_n \gg \Delta f_s h$, we have $T_\lambda/w \approx \int_{-a}^{a} \bar{f}_n s ds$. Thus, the torque is controlled by $\bar{f}_n$. Since $\bar{f}_n$ is independent of λ, the the total hydrodynamic torque is also independent of λ at the leading order in b when $\alpha = \pi/2$.

Case $\alpha = 0$**.** Proceeding as for the case $\alpha = \pi/2$ we get

$$\dot{\gamma}\eta h = h\Delta f_s + \lambda\frac{4ha}{\pi(a^2-s^2)}\Delta f_s + h\epsilon_s, \tag{28}$$

$$0 = -2\int_{-a}^{a}\left[\bar{f}_n(s_1)\ln(s'^2+4h'^2)-\partial_{s_1}(h(s_1)\Delta f_s(s_1))\ln s'^2\right]\mathrm{d}s_1+\frac{h\Delta f_s}{2\pi}\int_{-a}^{a}\frac{1}{s'}\mathrm{d}s_1+h\epsilon_n. \tag{29}$$

Equating the $O(h)$ terms gives $f_n \sim O(b)$, independent of λ. For the tangential traction we get

$$\Delta f_s \approx \frac{1}{1+4\lambda a/(\pi(a^2-s^2))}\left(\dot{\gamma}\eta - \epsilon_s(s,\lambda)\right). \tag{30}$$

As shown in **Supplementary Figure 5 b**, the decrease of Δf_s with λ/a predicted by the BI simulations is consistent with Eq. (30). As λ/a increases, the difference between the simulation results and Eq. (30) increases, due to contributions from $\boldsymbol{\epsilon}$ becoming larger as λ increases. As $\lambda/a \to \infty$ the term Δf_s decreases as $\Delta f_s \sim a/\lambda$ while $\bar{f}_n$ is independent of λ to leading order. This decrease in Δf_s results in a positive (counter-clockwise) torque from the $\bar{f}_n s$ contribution. In the no-slip case, in contrast, $hf_s \simeq sf_n$, resulting in a small clockwise torque at $O(b^2)$. This small torque is responsible for the slow rotation predicted by Jeffery's theory for disk-like particles aligned with the flow [8].

Three-dimensional axisymmetric disk. The manuscript discusses simulation results for an object that is infinite in the z direction ("2D platelet"). The basic assumption, corroborated by prior work [8], is that the rotational behaviour of a thin platelet is, to leading order in b/a, essentially independent of the shape in the z direction. Here we extend our results to a three-dimensional geometry that varies in the z direction, by showing that the total torque applied on an axi-symmetric disk of radius a and half-thickness $b \ll a$ ("3D disk") aligned with the flow changes sign when λ reaches a value of the order of b.

The surface of the disk is parametrised using polar coordinates (θ, ϕ) in the $\hat{\boldsymbol{e}}_x - \hat{\boldsymbol{e}}_z$ plane. The corresponding Cartesian coordinates are $s = \rho\cos\theta$ and $z = \rho\sin\theta$, respectively. The thickness of the platelet is described by the function $h(\rho)$. For a thin axisymmetric body, Singh et al. [8] verified that $f_s \approx f_1(\rho)$, $f_n \approx f_2(\rho)\cos\theta$ and $f_z \approx 0$. Thus Eq. (10:16) are also valid for the axisymmetric disk except that the following 3D Green's functions are required:

$$G_{ij}(s,h) = (8\pi)^{-1}\left(\partial_{ij}/(s^2+h^2)^{1/2} + 2x_ix_j/(s^2+h^2)^{3/2}\right), \quad K_{ijk}(s,h) = -(3/4\pi)\left(x_ix_jx_k/(s^2+h^2)^5\right). \tag{31}$$

In the region away from the edge, where the difference between ρ and ρ_1 is $\rho'' = |\rho_1 - \rho| \sim O(b)$, we have $h(\rho) \approx h(\rho_1)$, $\Delta f_s(\rho) \approx \Delta f_s(\rho_1)$ and these quantities are all constant to leading order, as for the 2D platelet. The expansions for S_s and S_n are [8]:

$$S_s = -h\Delta f_s(\rho) + O(b^2), \quad S_n = -2h\int_{S^+}\left[\frac{1}{\rho''}\left(\bar{f}_n(\rho_1)+\cos\theta_1\partial_{\rho_1}\left(h(\rho_1)\Delta f_s(\rho_1)\right)\right)\right]dS_1 + O(b^2). \tag{32}$$

To leading order, the equation for S_s is identical to Eq. (21) for an ellipse. Also, the equation for S_n is the same as Eq. (22), up to an logarithmic correction. The corresponding approximations for $K_s[\lambda\Delta f_s/\eta, 0, \lambda]$ =

$(\lambda/\eta)\int_{-a}^{a}\Delta f_s K_{ssn}^{+}\mathrm{d}s_1$ and $K_n[\lambda\Delta f_s/\eta, 0, \lambda] = (\lambda/\eta)\int_{-a}^{a}\Delta f_s K_{nns}^{+}\mathrm{d}s_1$ can be calculated similarly, using Eq. (20). Because $\rho'' \sim O(b)$ we have $dS_1 \approx \rho'' d\rho'' d\theta''$ with $\theta'' = \theta_1 - \theta$. The leading equations for K_s and K_n are thus:

$$K_s = \frac{\lambda}{\eta}\int_{S^+}\Delta f_s(\rho_1)K_{ssn}^{+}dS_1 = \frac{\lambda}{\eta}\Delta f_s\int_0^a\int_0^{2\pi}\frac{6}{4\pi}\frac{h\rho''^3\cos\theta''^2}{(\rho''^2+4h^2)^{5/2}}d\theta'' d\rho'' + O(\lambda b^2) \quad (33)$$

$$= \frac{\lambda}{\eta}\Delta f_s\left[\frac{1}{2} - \frac{3}{2}\frac{h}{a}\right] + O(\lambda b^2). \quad (34)$$

and

$$K_n = \frac{\lambda}{\eta}\int_{S^+}\Delta f_s(\rho_1)K_{nns}^{+}dS_1 = \frac{\lambda}{\eta}\Delta f_s\int_0^a\int_0^{2\pi} -\frac{3}{\pi}\frac{h^2\rho''^2\cos\theta''}{(\rho''^2+4h^2)^{5/2}}d\theta'' d\rho'' + O(\lambda b^2) \sim O(\lambda b^2). \quad (35)$$

Substituting Eq. (32:35) into Eq. (10) and Eq. (13) for $\alpha = 0$ yields the following leading order expressions:

$$\dot\gamma\eta h = h\Delta f_s + \lambda\frac{3h}{2a}\Delta f_s, \quad (36)$$

$$0 = -2h\int_{S^+}\left[\frac{1}{\rho''}\left(\bar f_n(\rho_1) + \cos\theta_1\partial_{\rho_1}\left(h(\rho_1)\Delta f_s(\rho_1)\right)\right)\right]dS_1. \quad (37)$$

Equating the $O(h)$ terms gives $f_n \sim O(b)$, independent of λ. For the tangential traction we get

$$\Delta f_s \approx \frac{\dot\gamma\eta}{1 + 3\lambda/(2a)}. \quad (38)$$

This value is identical to the value of Δf_s obtained for an ellipse (Eq. (30)), if a numerical prefactor of $3/2$ is used in front of λ/a instead of the prefactor $4/\pi$.

To identify the value of the critical slip length needed for the torque to change sign, one must evaluate Δf_s to leading order for $\lambda/a \ll 1$. For $\lambda/a \ll 1$, $\Delta f_s \approx \dot\gamma\eta\,[1 + k(\lambda/a)]$, where $k = 3/2$ for a 3D disk or $k = 4/\pi$ for a 2D plate. In the manuscript we showed that the torque changes sign when $-\eta\dot\gamma\lambda k/a + \Delta f_s^2 \sim 0$. Here $\Delta f_s^2 \sim \eta\dot\gamma b/a$ represents the second-order torque [8]. Thus, for both a 2D platelet or a 3D disk, the torque changes sign when λ is of the order of b.

Supplementary Note 2

This supplementary note presents details concerning the rigid approximation.

The assumption of a rigid platelet in our model can be rationalised from simple scaling arguments involving the competition between viscous stresses and bending forces. When the particle is aligned with the flow at its stable orientation α_c the total normal viscous stress applied to the particle is approximately

$$\eta\dot{\gamma}\left[f_y(\alpha=0)+(b/a)^2 f_y(\alpha=\pi/2)\right], \tag{39}$$

where f_y is the local normal stress. The normal stress $f_y(\alpha=0)\sim\mathcal{O}(b/a)$ over the surface, and $f_y(\alpha=0)\sim\mathcal{O}(1)$ near the edges (in a region of size $\sim\mathcal{O}(b/a)$) (**Supplementary Figure 5**). Also, $f_y(\alpha=\pi/2)\sim\mathcal{O}(1)$ (Supplementary Note 1). Hence, the normal viscous force on a particle aligned with the flow as given by Eq. (39) is in fact $\sim\mathcal{O}(\eta\dot{\gamma}ba)$, much smaller than the force $\sim\mathcal{O}(\eta\dot{\gamma}a^2)$ expected for a particle oriented perpendicularly to the flow. The bending rigidity B scales approximately as $B\approx Db^3$ with $D\approx 10^{11}$ J/m^3 [9]. Equating the normal viscous force with the bending forces $\sim B/a$, one obtains the following condition for buckling:

$$\eta\dot{\gamma}(a/b)^2/D\sim 1. \tag{40}$$

Supplementary References

[1] Pozrikidis, C. *Boundary integral and singularity methods for linearized viscous flow* (Cambridge University Press, 1992).

[2] Bocquet, L. & Barrat, J.-L. Flow boundary conditions from nano-to micro-scales. *Soft matter* 685 (2007).

[3] Luo, H. & Pozrikidis, C. Interception of two spheres with slip surfaces in linear Stokes flow. *J. Fluid Mech.* **581**, 129 (2007).

[4] Luo, H. & Pozrikidis, C. Effect of surface slip on Stokes flow past a spherical particle in infinite fluid and near a plane wall. *J. Eng. Math.* **62**, 1 (2008).

[5] Chwang, A. T. & Wu, T. Y. T. Hydromechanics of low-Reynolds-number flow. Part 2. Singularity method for Stokes flows. *J. Fluid Mech.* **67**, 787 (1975).

[6] Pozrikidis, C. *A practical guide to boundary element methods with the software library BEMLIB* (CRC Press, 2002).

[7] Press, W. H. *Numerical recipes 3rd edition: The art of scientific computing* (Cambridge University Press, 2007).

[8] Singh, V., Koch, D. L., Subramanian, G. & Stroock, A. D. Rotational motion of a thin axisymmetric disk in a low Reynolds number linear flow. *Phys. Fluids* **26**, 033303 (2014).

[9] Lindahl, N. *et al.* Determination of the bending rigidity of graphene via electrostatic actuation of buckled membranes. *Nano Lett.* **12**, 3526 (2012).
