# Supplementary material for: Hydrodynamic slip can align thin nanoplatelets in shear flow
Source: Nat Commun. 2020 May 15;11:2425. doi: 10.1038/s41467-020-15939-w (PMC7229003; doi:10.1038/s41467-020-15939-w)
Supplement: Supplementary file 3 — Description of Additional Supplementary Files [file 41467_2020_15939_MOESM3_ESM.pdf]

Description of Additional Supplementary Files

Supplementary Movie 1

Freely suspended graphene bilayer in a shear flow of strength $\dot{\gamma}$ *as extracted from MD simulations.*
